# Acute Phase Protein Response and Polymorphonuclear Leukocyte Cathepsin G Release After Slow Interleukin-1 Stimulation in the Rat

**DOI:** 10.1155/S0962935194000608

**Published:** 1994

**Authors:** Peter Björk, Thorarinn Gudmundsson, Kjell Ohlsson

**Affiliations:** Department of Surgical Pathophysiology University of Lund Malmö General Hospital Malmö S-214 01 Sweden

## Abstract

In this work we have studied the acute phase protein response and degranulation of polymorphonuclear leukocytes *in vivo* in the rat after a slow interleukin-1β stimulation. A total dose of 1 μg, 2 μg, 4 μg and 0 μg (controls with only vehicle) of interleukin-1β was released from osmotic minipumps over a period of 7 days. The pumps were implanted subcutaneously. A cystic formation was formed around the pumps that contained interleukin-1β whereas no tissue reaction was seen around pumps containing only vehicle. Besides flbroblasts the cyst wall contained numerous polymorphonuclear leukocytes which were positively stained for cathespin G. α_2_-macroglobulin, α_1_-inhtbitor-3, α_1_-proteinase inhibitor, albumin and C3 were measured by electroimmunoassay and all showed plasma concentration patterns that were dose-dependent to the amount of interleuktn-1β released. Fibrinogen in plasma was elevated in the control group but showed decreased plasma values with higher doses of interleukin-1β released. All animals showed increased plasma levels of cathespin G but the lowest levels for cathespin G were seen for the highest interleukin-1β dose released. It was clearly seen that a slow continuous release of interleukin-1β *in vivo* caused an inflammatory reaction. Plasma levels for the proteins analysed all showed a similar pattern, namely an initial increase or decrease of plasma concentration followed by a tendency to normalization of plasma values. It was concluded that a long-term interleukin-1β release could not sustain the acute phase protein response elicited by the initial interleukin-1β release.


Mediators of Inflammation 3, 425–431 (1994)

### Acute phase protein response and polymorphonuclear leukocyte cathepsin G release after slow interleukin-1 stimulation in the rat

Peter Björk,[CA] Thorarinn Gudmundsson and Kjell Ohlsson

Department of Surgical Pathophysiology, University of Lund, Malmö General Hospital, S-214 01 Malmö, Sweden

[CA] Corresponding Author

**In this work we have studied the acute phase protein response and degranulation of polymorphonuclear leukocytes *in vivo* in the rat after a slow interleukin-1β stimulation. A total dose of 1μg, 2μg, 4μg and 0μg (controls with only vehicle) of interleukin-1β was released from osmotic minipumps over a period of 7 days. The pumps were implanted subcutaneously. A cystic formation was formed around the pumps that contained interleukin-1β whereas no tissue reaction was seen around pumps containing only vehicle. Besides fibroblasts the cyst wall contained numerous polymorphonuclear leukocytes which were positively stained for cathespin G. $\alpha_2$-macroglobulin, $\alpha_1$-inhibitor-3, $\alpha_1$-proteinase inhibitor, albumin and C3 were measured by electroimmunoassay and all showed plasma concentration patterns that were dose-dependent to the amount of interleukin-1β released. Fibrinogen in plasma was elevated in the control group but showed decreased plasma values with higher doses of interleukin-1β released. All animals showed increased plasma levels of cathespin G but the lowest levels for cathespin G were seen for the highest interleukin-1β dose released. It was clearly seen that a slow continuous release of interleukin-1β *in vivo* caused an inflammatory reaction. Plasma levels for the proteins analysed all showed a similar pattern, namely an initial increase or decrease of plasma concentration followed by a tendency to normalization of plasma values. It was concluded that a long-term interleukin-1β release could not sustain the acute phase protein response elicited by the initial interleukin-1β release.**

**Key words:** Acute phase response, $\alpha_1$-inhibitor-3, $\alpha_2$-macroglobulin, Cathespin G, C3, Interleukin-1, Osmotic mini pump, Polymorphonuclear leukocytes, Proteinase inhibitors, Proteinases

### Introduction

An inflammatory reaction may be defined as 'the reaction of vascularized living tissue to local injury'.[1] The local tissue injury may be caused by mechanical trauma, radiation, microorganisms (infection), neoplasia, burn injury, chemical irritation, etc. Regardless of how the local tissue injury is caused, the vascularized living tissue will react mainly in a uniform way. This is probably due to the different specialized inflammatory cells and their capability to communicate. By inflammatory mediators and cellular receptors, cells are signalling and receiving signals in a huge complex network. Interleukin-1 (IL-1)[2] is a well-known inflammatory mediator holding a central position in the inflammatory reaction. It has the capability to induce an acute phase protein response from the liver and to induce degranulation of polymorphonuclear leukocytes (PMNs) *in vivo*.[3] Several plasma proteins are increased or decreased as a part of the acute phase response and this acute phase protein response differs from species to species.[4] Two proteinase inhibitors, namely $\alpha_1$-antichymotrypsin and $\alpha_1$-proteinase inhibitor ($\alpha_1$PI), are among the strongest reacting acute phase proteins in humans. By forming complexes with PMN cathepsin G and elastase they inhibit these two PMN proteases.[5] In the rat $\alpha_2$-macroglobulin ($\alpha_2$M) and $\alpha_1$-inhibitor-3 ($\alpha_1 I_3$) are marked acute phase proteins.[6] The objective of the present work was to investigate the effects of a slow continuous stimulation of IL-1 on some acute phase proteins and PMNs degranulation *in vivo*.

### Materials and Methods

*Assays*: Rat cathepsin G was measured by a specific enzyme-linked immunosorbent assay (ELISA) as described.[3] Rat $\alpha_2$M, $\alpha_1 I_3$, $C_3$ fibrinogen, $\alpha_1$PI and albumin were measured by electroimmunoassay.[7] Antisera against rat $\alpha_2$M and $\alpha_1 I_3$ were prepared as described.[8,9] Rabbit anti-rat $C_3$ and rabbit anti-rat fibrinogen were obtained from Cappel, USA and rabbit anti-rat $\alpha_1$PI and rabbit anti-rat albumin were

© 1994 Rapid Communications of Oxford Ltd a gift from Dr C-B. Laurell (Department of Clinical Chemistry, Malmö General Hospital). Enzymatic activity of cathepsin G was determined using the substrate Suc–Ala–Ala–Pro–Phe–pNA (SucAAPP) (Sigma) as described.[10]

*Immunohistochemistry*: Formalin fixed tissue sections were embedded in paraffin, cut and mounted on glass slides. Staining was done by the peroxidase–antiperoxidase method as described.[11]

*Animal experiments*: Female Wistar rats (Møllegaard Avelslaboratorium A/S, DK-4623 Skensved, Denmark) weighing 230 g were used. The animals were anaesthetized by an intraperitoneal injection of Mebumal® and then the back of the animals were shaved and disinfected. Through a small incision (approximately 15 mm in length) in the skin an osmotic minipump 'Model 2001' (Alzet) was implanted subcutaneously. The flow rate of the pump was 1 µl/h for 7 days. After implantation the rats were allowed to wake up. Four groups of rats containing five animals each received pumps with different amounts of recombinant human IL-1β (rhIL-1β), 0 µg (controls), 1 µg, 2 µg and 4 µg. In addition, two rats received minipumps subcutaneously containing 4 µg IL-1. After 3 days these two latter pumps were removed and reimplanted in two new rats which were followed for 3 days in an attempt to test the functional stability of rhIL-1β during the experimental conditions. The rhIL-1β was prepared by recombinant DNA technology in *Escherichia coli* (Synergen Inc., USA). The protein was diluted to appropriate concentrations in sterile filtered phosphate buffered saline, pH 7.4 (Dulbecco) with 0.2% (w/v) bovine serum albumin (Sigma) and filled into the pumps. Blood samples of approximately 0.4 ml were taken from the tail into tubes containing EDTA. Plasma was immediately prepared and frozen at –70°C. Samples were collected at time 0, just before the implantation of the osmotic minipumps and thereafter every 24 h for 7 days. After 7 days the animals were sacrificed with an overdose of Mebumal® and the eventual cystic formation and cystic fluid around the pumps were collected. The cystic formations were fixed in phosphate buffered formalin until paraffin embedded and the cystic fluids were centrifuged and frozen at –70°C until analysed. During the whole experimental time the animals had free access to water and standard pellet

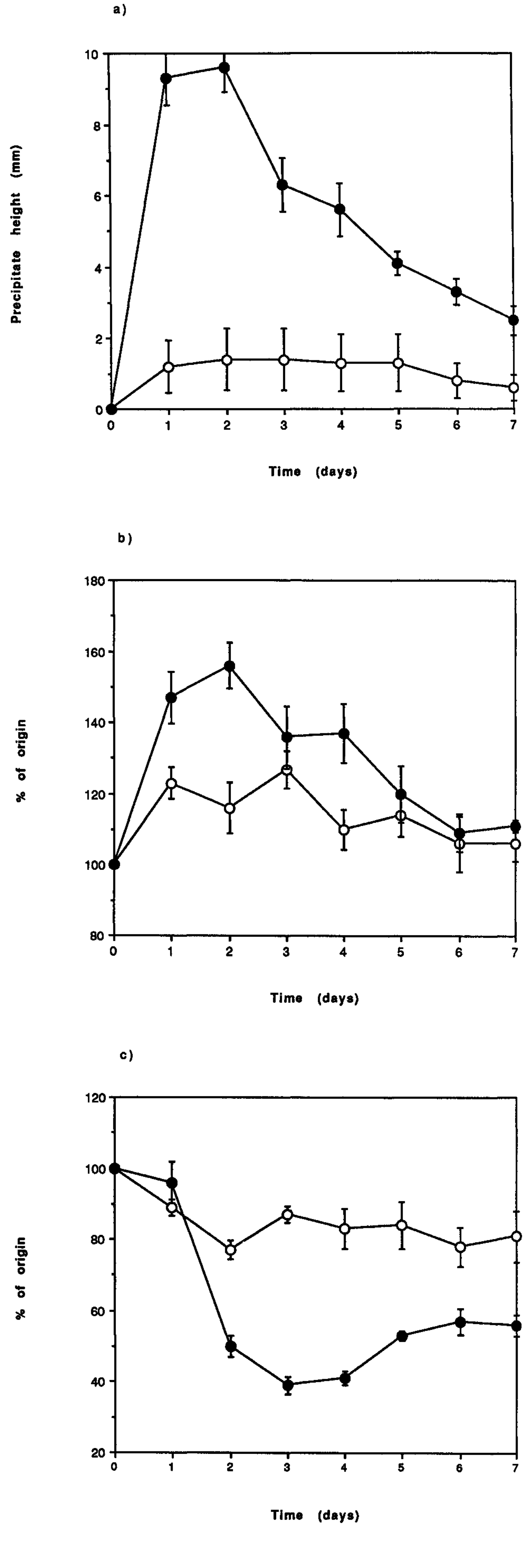

FIG. 1. Using subcutaneously implanted osmotic minipumps rats were stimulated by a slow release of different amounts of IL-1β diluted in 0.2% BSA (w/v) in PBS. One group received only vehicle (control group), while three other groups received 6 ng/h IL-1β, 12 ng/h IL-1β and 24 ng/h IL-1β, respectively. Here results from the control group (O-O) and the group that received 24 ng/h IL-1β (●-●) are presented. Blood samples were taken from the tail just before implantation of the osmotic pump and thereafter for 7 days. Values are given as mean values ± standard error of the mean. All groups contained five rats. $\alpha_2$M (a), $\alpha_1$PI (b), $\alpha_1I_3$ (c), albumin (d), $C_3$ (e) and fibrinogen (f) were measured by EIA. Plasma cathepsin G (g) was measured by a specific ELISA.

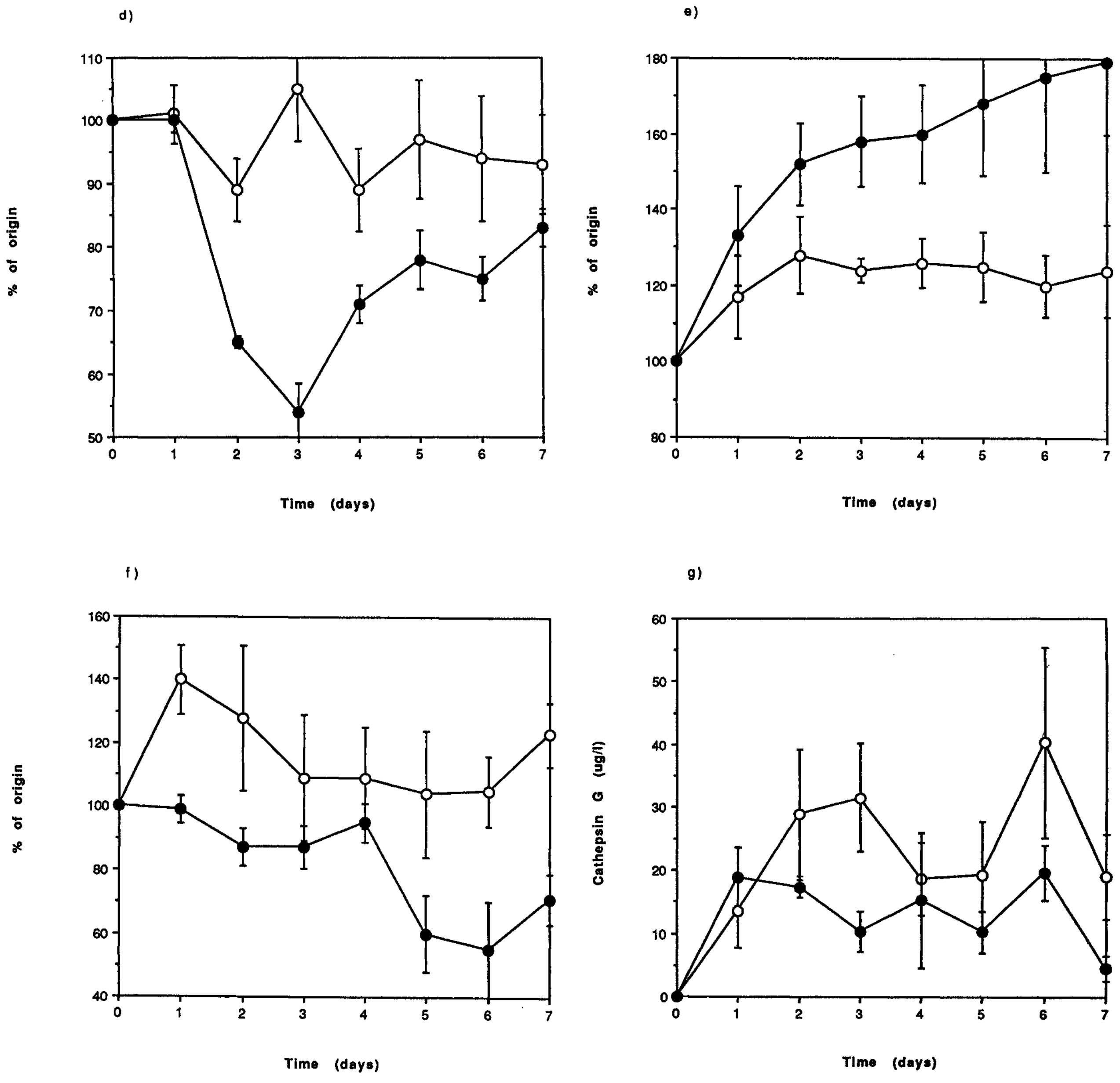

FIG. 1. *Continued.*

food. The animal experiments were sanctioned by the local ethical committee for animal experiments.

### Results

*Tissue reactions*: The rats did not show any outer signs of discomfort throughout the experiment. At autopsy a cystic formation was seen around osmotic minipumps containing IL-1 irrespective of dose, whereas no tissue reaction was seen around pumps without IL-1 (controls). No enzymatic activity of cathepsin G as measured by activity against SucAAPP could be detected in the cystic fluid. Cathepsin G concentration in the fluids was measured by a specific ELISA and mean value ± SEM in the groups were: 1 μg IL-1 = 179 ± 7 μg/l, 2 μg IL-1 = 179 ± 13 μg/l and 4 μg/l = 176 ± 8 μg/l.

*Acute phase proteins*: $\alpha_2$M, $\alpha_1$I$_3$, C$_3$, fibrinogen, $\alpha_1$PI and albumin were all measured by electroimmunoassay. $\alpha_2$M was not detectable in plasma at time 0, just before the implantation of the osmotic minipumps, when measured by electroimmunoassay. Detectable amounts were seen after one day and throughout the observation period with peak values on day 1 and 2 (Fig. 1a). A dose–response pattern was seen for $\alpha_2$M. Plasma $\alpha_1$PI showed the highest values on day 2 with values ranging from 140 to 160% of the starting levels (Fig. 1b). The values then gradually decreased during the experimental time. $\alpha_1$I$_3$ plasma levels decreased with the greatest decrease in the group that received 4 μg IL-1 (Fig. 1c). Normal plasma level was not totally restored during the observation period. The same pattern was seen for albumin (Fig. 1d). Plasma values for C3 were increased during the whole experimental time (Fig. 1e). Rats treated with the highest doses showed a value of 180% on day 7 compared with the starting value. After an initial increase of fibrinogen in the controls the values thereafter decreased. However, in the two groups receiving the largest amounts of IL-1 an almost immediate decrease in plasma values were seen (Fig. 1f). An equal increase in $\alpha_2$M plasma levels were seen in the two rats that received a pump formerly implanted for 3 days in two other rats (Fig. 2).

*Plasma cathepsin G in IL-1 stimulated rats*: Plasma levels of cathepsin G at time 0 were below the detection level 1.5 μg/l of the specific ELISA used. Irrespective of the IL-1 dose administered, a biphasic curve was seen in plasma levels (Fig. 1g).

*Immunohistochemistry*: In sections from the cystic formation with adjacent skin numerous inflammatory cells were seen in the dense wall of fibroblasts (Fig. 3a). Approximately half of the inflammatory cells were stained for cathepsin G.

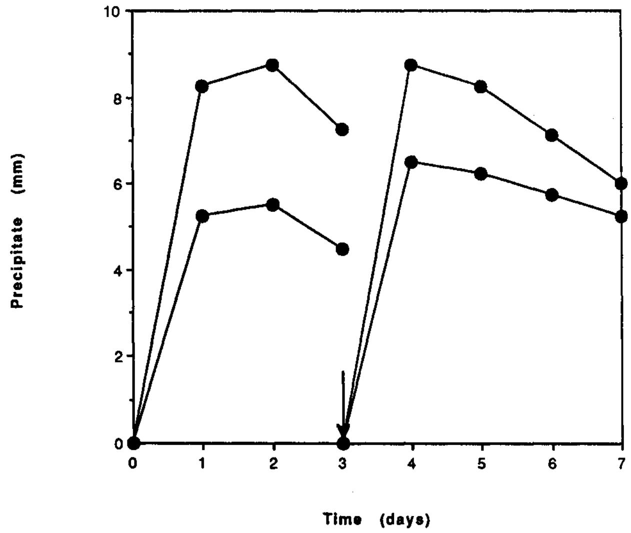

FIG. 2. Osmotic minipumps containing 4 μg IL-1β was implanted subcutaneously in two rats. Plasma samples were taken prior to implantation and thereafter daily for 3 days. After 3 days the pumps were removed and implanted in two new rats. Blood samples were taken prior to implantation of the pumps and thereafter daily for 4 days. Plasma levels of $\alpha_2$M are given (●–●). The arrow indicates the time for reimplantation in new rats.

### Discussion

IL-1 is an important cytokine released in the inflammatory process. There are two forms of IL-1, IL-1α and IL-1β.[2] They both act on the same receptors[12] and they also elicit the same cellular response. Recently a specific IL-1 receptor antagonist (IL-1ra) has been isolated in its native form and also cloned.[13–15] The importance of IL-1 in the acute inflammatory process a

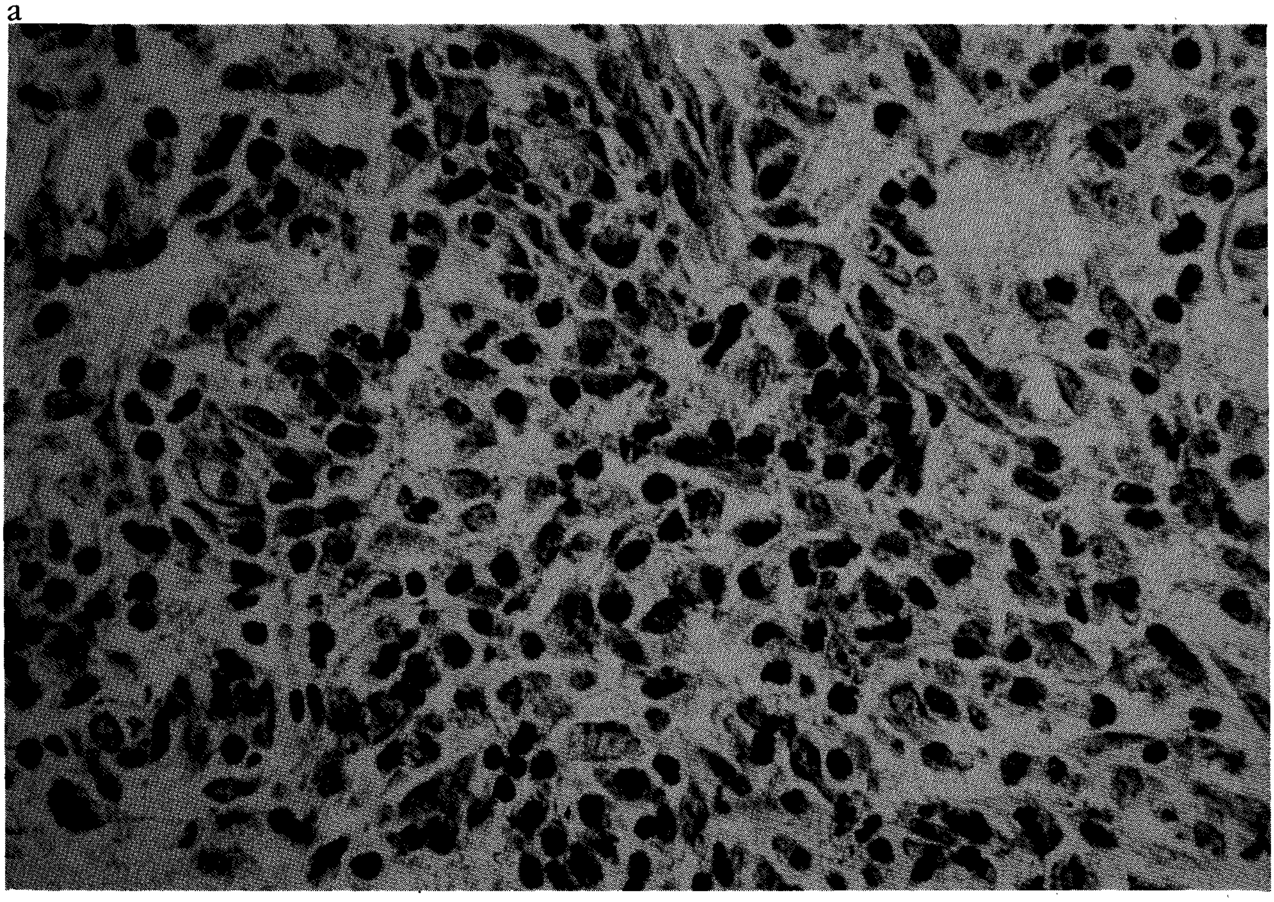

FIG. 3. Section from cyst formation formed around osmotic minipumps containing IL-1β. (a) Hematoxylin eosin stained tissue, magnification × 500. Numerous inflammatory cells are seen in the wall of the cyst. (b) Tissue only immunohistochemically stained with rabbit anti-rat PMN cathepsin G, magnification × 500. Note PMNs containing cathepsin G. (c) Tissue incubated with normal rabbit serum as control.

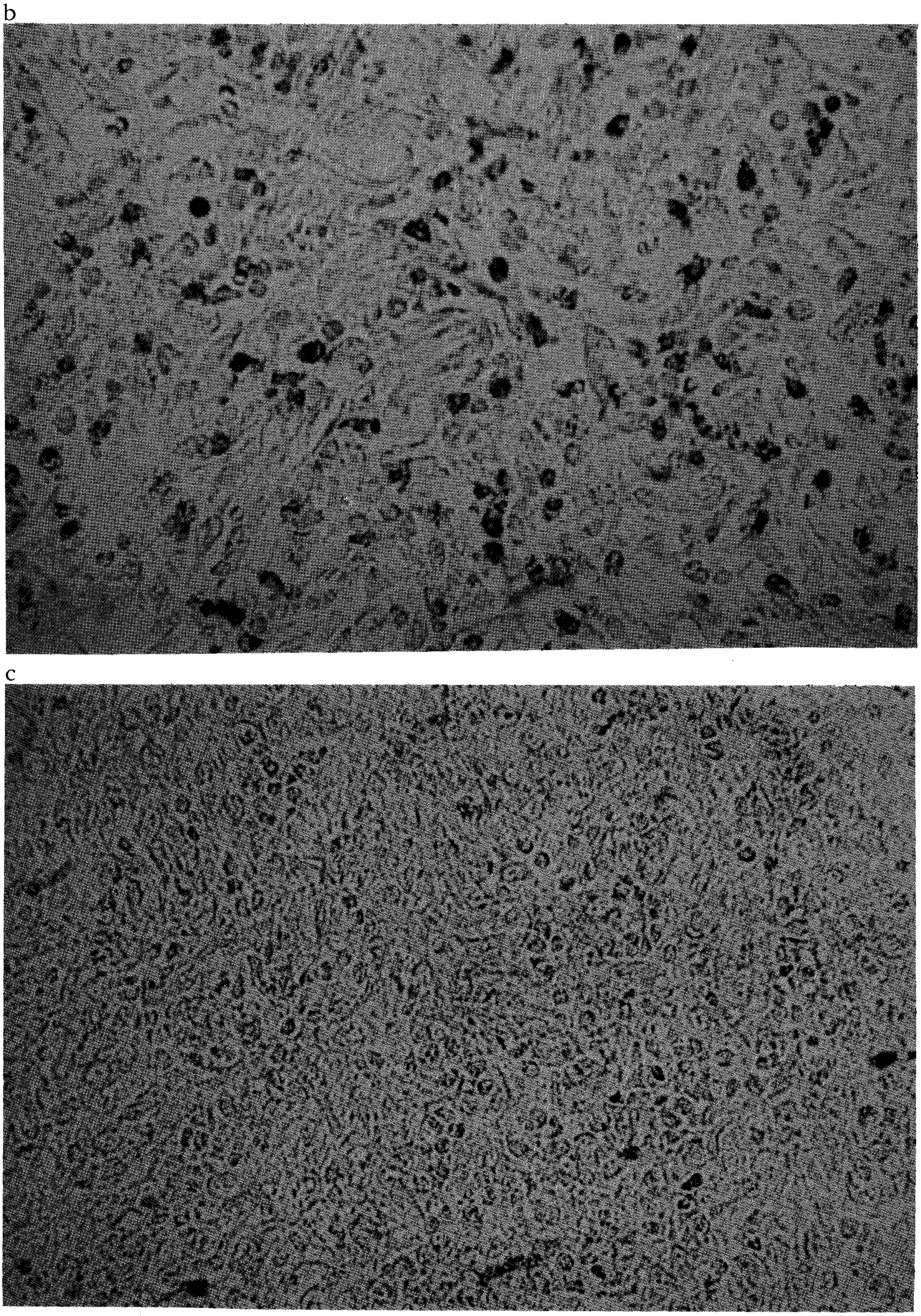

FIG. 3. *Continued.*

may be illustrated by survival of rabbits receiving a normally lethal endotoxin dose after treating the animals with intravenous injections of IL-1ra.[16] Although the effects of IL-1 are well documented most of the knowledge has come from studies *in vitro* and studies *in vivo* may be a valuable complement for the understanding of the pathophysiological role of IL-1.

In this work we have studied the effects on the acute phase protein response and the degranulation of PMNs by a slow IL-1 release stimulation over a period of 7 days. Even with only vehicle present in the mini osmotic pumps (control group) a clear inflammatory reaction could be seen for the acute phase proteins. In these animals fibrinogen, $\alpha_1$PI and $\alpha_2$M all showed elevation of plasma values for the first 2 days but these values decreased over time towards the origin values. C3 in plasma increased to 125% after 2 days and thereafter the plasma values stayed at this level throughout the observation time. Both albumin and $\alpha_1 I_3$ showed decreased plasma values over the whole observation period. Cathepsin G was released to the bloodstream as measured by elevated plasma levels from the second day to the seventh day. All these findings are in agreement with an inflammatory reaction due to the trauma from the operative procedure. No local tissue reaction was seen around the implanted pumps only containing vehicle, indicating that no greater irritation was caused to the surrounding tissue.

When IL-1 was released from the mini osmotic pumps a more pronounced acute phase protein reaction was seen. For $\alpha_2$M, $\alpha_1$PI and C3 there was a dose-dependent pattern seen in the elevated plasma values. Also for the negative acute phase protein reactants albumin and $\alpha_1 I_3$ a dose-dependent pattern could be detected. Fibrinogen is a positive acute phase protein reactant in humans reaching a maximum plasma concentration approximately on the third day after tissue injury.[17] When increasing amounts of IL-1 were given the plasma levels of fibrinogen decreased over time and the positive acute phase response was changed into a negative acute phase response. How this change in acute phase answer is mediated is not known but our results are in agreement with a study by De Jong *et al.*[18]

The data so far discussed clearly show that the tissue injury caused by implantation of the osmotic minipump is causing an acute phase protein response and that this response is enhanced by the stimulation of IL-1β. The general plasma protein levels over the experimental time showed an initial peak or dip followed by a tendency to return to original values in spite of the continuous release of IL-1 over the whole experimental time. To examine that the IL-1 in the pumps did not lose its biological activity we removed pumps from two rats and reimplanted them in two new rats. The same reaction pattern for $\alpha_2$M was seen in all these four rats clearly indicating that IL-1 did not lose its biological activity over the experimental time. Therefore, an explanation for these results may be that the initial release of IL-1 also provokes a reaction to counteract effects of IL-1. This effect could be mediated by an induction of IL-1 receptor antagonist. IL-1 has the capability to induce IL-6[19] production which in turn may induce IL-1 receptor antagonist production as recently shown.[20] Another explanation could be inactivation of IL-1 in the immediate surrounding of the pumps by proteolytic enzymes. In this study we showed high levels of cathepsin G in the cystic formations. However, no enzymatic activity against the cathepsin G substrate SucAAPP was detected indicating the measured levels of cathepsin G to be enzymatically inactive probably by inhibition by protease inhibitors or by autodigestion.

Around the osmotic mini pumps cystic formations containing a serous liquid were formed when the pumps contained IL-1, while in pumps with only vehicle no cystic formation was seen. This reaction has previously been described by Lewis *et al.*[21] and Dunn *et al.*[22] It is known that IL-1 has the capability to stimulate fibroblasts[23] and IL-1 is therefore suggested to be one of the major mediators of skin inflammation. It is likely that the cystic formation seen is a result of such a stimulation. Cathepsin G was measured in the liquids and values were approximately 170 g/l irrespective of the IL-1 dose given. In the wall of the cystic formation numerous inflammatory cells could be seen in histological sections. About 50% of the cells were stained for cathepsin G which may be a source for the high concentrations of cathepsin G seen in the fluid. Cathepsin G and other proteases like elastase from PMNs have the capability to degrade proteins and cathepsin G and elastase has been shown to be able to degrade TNFα and TNFβ but not IL-1α.[24] The presence of proteases in this cystic formation surrounding the minipump may influence the IL-1 released.

While the acute phase proteins showed a dose-dependent response to the amount of IL-1 administered cathepsin G plasma levels did not show any tendency to a dose-dependent release. These data are in contrast to our previous study where cathepsin G plasma levels showed a dose-dependent pattern to a single injection of IL-1.[3] We have no obvious explanation for the difference in these results. When proteolytic enzymes like cathepsin G are released to the bloodstream, complexes with inhibitors are formed. These complexes are then removed from the circulation by the liver. This removal is mediated by specific receptors on the endothelial cells in the liver.[25] It may be speculated that the capacity of the liver to remove complexes from the bloodstream may be improved due to the long standing IL-1

stimulation and thus the lowest cathepsin G plasma concentrations are measured in the highest IL-1 dose given.

To summarize, we have found the initial release of IL-1 to be the most important stimulation regarding the acute phase protein response from the liver. Although the IL-1 release was continued for 7 days the acute phase protein levels in plasma approached towards normal values after the increase/decrease during the first 2 days.

### References

1. Cotran RS, Kumar V, Robbins SL. In: *Pathologic Basis of Disease* (4th ed). W.B. Saunders Company, Philadelphia, PA, 1989; 39.
2. Dinarello CA. Interleukin-1 and its biologically related cytokines. *Adv Immunol* 1989; **44**: 153–205.
3. Björk P, Ohlsson K. The interleukin-1 receptor antagonist influences interleukin-1 effects in rat and mouse. *Med Infl* 1992; **1**: 27–31.
4. Koj A, Magielska-Zero D, Kurdowska A, Bereta J. Proteinase inhibitors as acute phase reactants: regulation of synthesis and turnover. *Adv Exp Med Biol* 1988; **240**: 171–181.
5. Travis J. Structure, function, and control of neurophil proteinases. *Am J Med* 1988; **84** (suppl 6A): 37–42.
6. Lonberg-Holm K, Reed DL, Roberts RC, Herbert RR, Hillman MC, Kutney RM. Three high molecular weight protease inhibitors of rat plasma. *J Biol Chem* 1987; **262**: 438–445.
7. Laurell CB. Electroimmuno assay. *Scand J Clin Lab Invest* 1972; **29** (suppl 124): 21–37.
8. Gauthier F, Mouray H. Rat $\alpha_2$ acute-phase macroglobulin isolation and physicochemical properties. *Biochem J* 1976; **159**: 661–665.
9. Gauthier F, Ohlsson K. Isolation and some properties of a new enzyme-binding protein in rat plasma. *Hoppe-Seyler's Z Physiol Chem* 1978; **359**: 987–992.
10. Nakajima K, Powers JC, Ashe BM, Zimmerman M. Mapping the extended substrate binding site of cathepsin G and human leukocyte elastase. *J Biol Chem* 1979; **254**: 4027–4032.
11. Sternberger LA, Hardy Jr PH, Cuculis JJ, Meyer HG. The unlabelled antibody enzyme method of immunochemistry, preparation and properties of soluble antigen–antibody complex (horseradish peroxidase–antihorseradish peroxidase) and its use in identification of Spirochetes. *J Histochem Cytochem* 1970; **18**: 315–333.
12. Granowitz EV, Clark BD, Mancilla J, Dinarello CA. Interleukin-1 receptor antagonist competitively inhibits the binding of interleukin-1 to the type II interleukin-1 receptor. *J Biol Chem* 1991; **266**: 14147–14150.
13. Hannum CH, Wilcox CJ, Arend WP, *et al.* Interleukin-1 receptor antagonist activity of a human interleukin-1 inhibitor. *Nature* 1990; **343**: 336–340.
14. Eisenberg SP, Evans RJ, Arend WP, *et al.* Primary structure and functional expression from complementary DNA of a human interleukin-1 receptor antagonist. *Nature* 1990; **343**: 341–346.
15. Carter DB, Deibel Jr MR, Dunn CJ, *et al.* Purification, cloning, expression and biological characterization of an interleukin-1 receptor antagonist protein. *Nature* 1990; **344**: 633–638.
16. Ohlsson K, Björk P, Bergenfeldt M, Hageman R, Thompson RC. Interleukin-1 receptor antagonist reduces mortality from endotoxin shock. *Nature* 1990; **348**: 550–552.
17. Kampschmidt RF, Fuller GM. The effects of leucocyte endogenous mediator on plasma fibrinogen and haptoglobin. *Proc Soc Exp Biol Med* 1974; **146**: 904–907.
18. De Jong FA, Birch HE, Schreiber G. Effect of recombinant interleukin-1 on mRNA levels in rat liver. *Inflammation* 1988; **12**: 613–617.
19. Zoja C, Wang JM, Bettoni S, *et al.* Interleukin-1β and tumor necrosis factor-α induce gene expression and production of leukocyte chemotactic factors, colony-stimulating factors, and interleukin-6 in human mesangial cells. *Am J Pathol* 1991; **138**: 991–1003.
20. Tilg H. Trehu E. Atkins MB, Dinarello CA, Mier JW. Interleukin-6 (IL-6) as an anti-inflammatory cytokine: induction of circulating IL-1 receptor antagonist and soluble tumor necrosis factor receptor p55. *Blood* 1994; **83**: 113–118.
21. Lewis EJ, Sedgwick AD, Hanahoe THP. *In vivo* changes in plasma acute phase protein levels in the rat induced by slow release of IL-1, IL-6 and TNF. *Med Infl* 1992; **1**: 39–44.
22. Dunn CJ, Hardee MM, Staite ND. Acute and chronic inflammatory responses to local administration of recombinant IL-1α, IL-1β, TNFα, IL-2 and IFNγ in mice. *Agents & Actions* 1989; **27**: 290–293.
23. Croute F, Delaporte E, Bonnefoy JY, Fertin C, Thivolet J, Nicolas JF. Interleukin 1β stimulates fibroblast elastase activity. *Br J Dermatol* 1991; **124**: 538–541.
24. Scuderi P, Nez A, Duerr ML, Wong BJ, Waldes CM. Cathepsin-G and leukocyte elastase inactivate human tumor necrosis factor and lymphotoxin. *Cell Immunol* 1991; **135**: 299–313.
25. Pizzo SV, Mast AE, Feldman SR, Salvesen G. *In vivo* catabolism of $\alpha_1$-antichymotrypsin is mediated by the Serpin receptor which binds $\alpha_1$-proteinase inhibitor, antithrombin III and heparin cofactor II. *Biochim Biophys Acta* 1988; **967**: 158–162.

ACKNOWLEDGEMENTS. We wish to thank Iréna Ljungkrantz for excellent technical assistance and Dr R. C. Thompson, Synergen Inc., for providing human recombinant interleukin-1β.

This work was supported by research funds from: The Swedish Medical Research Council (3910); The Medical Faculty, University of Lund; The Swedish Medical Society; The Albert Påhlsson Foundation, The Foundation for Cancer Research administered by Malmö General Hospital, Alfred Österlund Foundation and Greta and Johan Kocks Foundations.

**Received 4 July 1994; accepted in revised form 15 July 1994**

## References

[PDF_00410] Bjrök P., Ohlsson K. (1992). The interleukin-1 receptor antagonist influences interleukin-1 effects in rat and mouse.. Mediators Inflamm.

[PDF_00442] Carter D. B., Deibel M. R., Dunn C. J., Tomich C. S., Laborde A. L., Slightom J. L., Berger A. E., Bienkowski M. J., Sun F. F., McEwan R. N. (1990). Purification, cloning, expression and biological characterization of an interleukin-1 receptor antagonist protein.. Nature.

[PDF_00465] Croute F., Delaporte E., Bonnefoy J. Y., Fertin C., Thivolet J., Nicolas J. F. (1991). Interleukin-1 beta stimulates fibroblast elastase activity.. Br J Dermatol.

[PDF_00450] De Jong F. A., Birch H. E., Schreiber G. (1988). Effect of recombinant interleukin-1 on mRNA levels in rat liver.. Inflammation.

[PDF_00408] Dinarello C. A. (1989). Interleukin-1 and its biologically related cytokines.. Adv Immunol.

[PDF_00462] Dunn C. J., Hardee M. M., Staite N. D. (1989). Acute and chronic inflammatory responses to local administration of recombinant IL-1 alpha, IL-beta, TNF alpha, IL-2 and Ifn gamma in mice.. Agents Actions.

[PDF_00439] Eisenberg S. P., Evans R. J., Arend W. P., Verderber E., Brewer M. T., Hannum C. H., Thompson R. C. (1990). Primary structure and functional expression from complementary DNA of a human interleukin-1 receptor antagonist.. Nature.

[PDF_00422] Gauthier F., Mouray H. (1976). Rat alpha2 acute-phase macroglobulin. Isolation and physicochemical properties.. Biochem J.

[PDF_00424] Gauthier F., Ohlsson K. (1978). Isolation and some properties of a new enzyme-binding protein in rat plasma.. Hoppe Seylers Z Physiol Chem.

[PDF_00434] Granowitz E. V., Clark B. D., Mancilla J., Dinarello C. A. (1991). Interleukin-1 receptor antagonist competitively inhibits the binding of interleukin-1 to the type II interleukin-1 receptor.. J Biol Chem.

[PDF_00437] Hannum C. H., Wilcox C. J., Arend W. P., Joslin F. G., Dripps D. J., Heimdal P. L., Armes L. G., Sommer A., Eisenberg S. P., Thompson R. C. (1990). Interleukin-1 receptor antagonist activity of a human interleukin-1 inhibitor.. Nature.

[PDF_00448] Kampschmidt R. F., Upchurch H. F. (1974). Effect of leukocytic endogenous mediator on plasma fibrinogen and haptoglobin.. Proc Soc Exp Biol Med.

[PDF_00420] Laurell C. B. (1972). Electroimmuno assay.. Scand J Clin Lab Invest Suppl.

[PDF_00459] Lewis E. J., Sedgwick A. D., Hanahoe T. H. (1992). In vivo changes in plasma acute phase protein levels in the rat induced by slow release of IL-1, IL-6 and TNF.. Mediators Inflamm.

[PDF_00417] Lonberg-Holm K., Reed D. L., Roberts R. C., Hebert R. R., Hillman M. C., Kutney R. M. (1987). Three high molecular weight protease inhibitors of rat plasma. Isolation, characterization, and acute phase changes.. J Biol Chem.

[PDF_00426] Nakajima K., Powers J. C., Ashe B. M., Zimmerman M. (1979). Mapping the extended substrate binding site of cathepsin G and human leukocyte elastase. Studies with peptide substrates related to the alpha 1-protease inhibitor reactive site.. J Biol Chem.

[PDF_00445] Ohlsson K., Björk P., Bergenfeldt M., Hageman R., Thompson R. C. (1990). Interleukin-1 receptor antagonist reduces mortality from endotoxin shock.. Nature.

[PDF_00470] Pizzo S. V., Mast A. E., Feldman S. R., Salvesen G. (1988). In vivo catabolism of alpha 1-antichymotrypsin is mediated by the Serpin receptor which binds alpha 1-proteinase inhibitor, antithrombin III and heparin cofactor II.. Biochim Biophys Acta.

[PDF_00467] Scuderi P., Nez P. A., Duerr M. L., Wong B. J., Valdez C. M. (1991). Cathepsin-G and leukocyte elastase inactivate human tumor necrosis factor and lymphotoxin.. Cell Immunol.

[PDF_00429] Sternberger L. A., Hardy P. H., Cuculis J. J., Meyer H. G. (1970). The unlabeled antibody enzyme method of immunohistochemistry: preparation and properties of soluble antigen-antibody complex (horseradish peroxidase-antihorseradish peroxidase) and its use in identification of spirochetes.. J Histochem Cytochem.

[PDF_00456] Tilg H., Trehu E., Atkins M. B., Dinarello C. A., Mier J. W. (1994). Interleukin-6 (IL-6) as an anti-inflammatory cytokine: induction of circulating IL-1 receptor antagonist and soluble tumor necrosis factor receptor p55.. Blood.

[PDF_00452] Zoja C., Wang J. M., Bettoni S., Sironi M., Renzi D., Chiaffarino F., Abboud H. E., Van Damme J., Mantovani A., Remuzzi G. (1991). Interleukin-1 beta and tumor necrosis factor-alpha induce gene expression and production of leukocyte chemotactic factors, colony-stimulating factors, and interleukin-6 in human mesangial cells.. Am J Pathol.

